# Postoperative Craniotomy Pain in Emergent versus Non-emergent Cases

**DOI:** 10.7759/cureus.5525

**Published:** 2019-08-29

**Authors:** Stephen Albano, Syed A Quadri, Mujtaba Ajaz, Yasir R Khan, Javed Siddiqi

**Affiliations:** 1 Neurosurgery, Desert Regional Medical Center, Palm Springs, USA; 2 Neurosurgery, California Institute of Neurosciences, Thousand Oaks, USA

**Keywords:** craniotomy, pain control

## Abstract

Background: Postoperative pain control in craniotomies poses multiple challenges. Pain must be addressed, but the use of medications must be weighed against risks. Craniotomies risk neurologic injury and so postoperative examinations are critical. Medications used to address pain can alter the neurological examination or cause bleeding leading to misdiagnosis of complications.

Objective: Determine if there is a significant difference in postoperative pain from emergent craniotomies vs. non-emergent craniotomies

Methods: A retrospective review included 102 cases performed from 2010-2016; pain scores were compared on post-operative days one, two, and three between emergent and non-emergent craniotomies.

Results: Pain scores for emergent cases on post-operative days one through three were 5.1 (standard deviation (SD)=2.9), 5.9 (SD=2.1), 4.7 (SD=3.0) respectively. Pain scores for non-emergent cases on post-operative days one through three were 5.7 (SD=2.6), 4.8 (SD=2.8), and 4.6 (SD=3.0) respectively. A one-way analysis of variance (ANOVA) was conducted to compare pain scores between groups for each post-operative day. On post-operative day, one there was no significant difference between the groups [F(1,100)=0.49, p=0.485]. On post-operative day two, there was no significant difference between the groups [F(1,100)=2.17, p=0.143]. On post-operative day three, there was no significant difference between the groups [F(1,98)=0.002, p=0.957].

Conclusion: There is no significant difference in the level of pain on postoperative days one through three between emergent and non-emergent craniotomy patients.

## Introduction

Postoperative pain control in craniotomies has been a challenge for years. Research has been directed at trying to identify improved analgesic regimens [1-9]. These papers have identified different regimens, each with benefits and risks. However, in general, research suggests that postoperative craniotomy pain is under treated [10-15] with an incidence of moderate to severe acute pain reported from 55%-70% [8,10,13-14]. Pain control is often difficult due to multimodal etiology in addition to concerns over medications risks in neurosurgical patients. Opioids are a powerful analgesic; however, often underutilized due to concerns of impairing the neurological examination due to changes in mental status and effects on cranial nerve function such as miosis which could mask catastrophic complications, and possibly lead to respiratory depression [1,3,5-7,11,15-17]. Analgesics such as non steroidal anti-inflammatory drugs (NSAIDs) can lead to impaired platelet function with the risk for postoperative bleeding [6-7,15-18]. Despite these risks, patients must still be treated for pain, therefore, an understanding of the patient’s pain post operatively and the risks associated with various analgesics are essential. Pain management strategies also include scalp blocks which have been shown to decrease the severity of pain post operatively [8]. This study was designed to determine if there is a difference between post-operative pain experienced by patients undergoing emergent craniotomy (within 24 hours of presentation) vs. non-emergent craniotomy. Identifying if there is a difference would allow better-informed decisions when managing patients post operatively.

## Materials and methods

A retrospective study at a single institution of all patients undergoing craniotomy from January 2010 to December 2016 were assessed for postoperative pain as recorded by nursing staff during medication administration for the first three postoperative days using the visual analog scale. Scores were averaged over each day for post-operative days one, two, and three. Inclusion criteria included craniotomy performed at the hospital of study and age greater than 18 years old. Exclusion criteria included the inability to communicate pain score verbally either through hand gestures or through writing, previous craniotomy or craniectomy, pregnancy, prison inmate, or incomplete records (for example missing recorded pain scores).

## Results

After a review of 939 potential records, 102 were included in the study after inclusion and exclusion criteria were applied. The average age of patients was 53 and approximately 53% of study participants were male (n=54) with 47% female (n=48). Reasons for surgery include tumor resection 70% (n=71), intracranial hemorrhage including epidural, subdural, or intraparenchymal 21% (n=21), arteriovascular malformation (AVM) or aneurysm 6% (n=6), or other 4% (n=4). Cases that satisfied criteria were largely non-emergent at 87% (n=89) and 13% emergent (n=13). Demographic information is shown in Table 1.

**Table 1:** Demographics and indications for surgery AVM - arteriovascular malformation

Population	Number of patients
Average age	53
Age range	18-84
Male	54 (53%)
Female	48 (47%)
Tumor resection	71 (70%)
Evacuation epidural, subdural, or intraparenchymal hematoma	21 (21%)
Vascular (AVM, aneurysm)	6 (6%)
Other	4 (4%)
Emergent (surgery <24 hours from presentation)	13 (13%)
Non emergent	89 (87%)

Aggregate pain scores of both emergent and non-emergent groups from post-operative days one to three scored 5.6 (standard deviation (SD)=2.7), 4.9 (SD+2.7), and 4.6 (SD=3.0), respectively. Pain scores for emergent cases on post-operative days one through three were 5.1 (SD=2.9), 5.9 (SD=2.1), 4.7 (SD=3.0), respectively. Pain scores for non-emergent cases on post-operative days one through three were 5.7 (SD=2.6), 4.8 (SD=2.8), and 4.6 (SD=3.0), respectively. Average pain scores are shown in Table 2 for visual comparison.

**Table 2:** Average pain score on indicated post operative day

Post Operative Day (POD)	Average score (standard deviation)
POD#1 emergent	5.1 (SD=2.9)
POD#1 non emergent	5.7 (SD=2.6)
POD#2 emergent	5.9 (SD=2.1)
POD#2 non emergent	4.8 (SD=2.8)
POD#3 emergent	4.7 (SD=3.0)
POD#3 non emergent	4.6 (SD=3.0)

A one-way analysis of variance (ANOVA) was conducted to compare pain scores in patients who underwent emergent craniotomy versus non-emergent craniotomy for each post-operative day. On post-operative day one, there was no significant difference between the groups [F(1,100)=0.49, p=0.485]. On post-operative day two, there was no significant difference between the groups [F(1,100)=2.17, p=0.143]. On post-operative day three, there was no significant difference between the groups [F(1,98)=0.002, p=0.957]. Post-operative day three has fewer data points as one patient was intubated and another taken back to the operating room for recurrent subdural hematoma. Regarding opioids, the majority of patients 95% (n=97) received some form of opioid for pain control with only 1% (n=1) of all patients returning to the operating room for recurrent subdural hematoma. ANOVA scores are shown in Table 3.

**Table 3:** Analysis of variance (ANOVA) for each post operative day

Post Operative Day (POD)	ANOVA
POD#1	F(1,100)=0.49, p=0.485
POD#2	F(1,100)=2.17, p=0.143
POD#3	F(1,98)=0.002, p=0.957

## Discussion

Pain scores in emergent versus non-emergent craniotomy cases did not show a statistically significant difference which could suggest that post-operative pain is dominated by surgical disruption of anatomy as opposed to the mechanism of injury. However, this study includes a disproportionate amount of non-emergent cases since emergent cases are likely left intubated or are unable to communicate pain effectively post operatively. Therefore, this study population likely represents less severe mechanisms of injury since they were able to be extubated post operatively. Scalp nerve blocks were inconsistently used in non-emergent cases with infiltration typically only taking place along the planned site of incision primarily to aid with hemostasis. Therefore, this could be a potential confounder in post operative pain score comparison. Trauma patients included in the study population may also have had additional injuries such as fractured bones which would confound the reported pain scores. Also, the perception of pain by each individual is different which will lead to significant variability. The averages of all patients for each group were computed and the variability is partially reflected in the standard deviation. Emergent craniotomies represent 13% of the study population and non-emergent craniotomies representing 87% which also makes comparison difficult due to the disproportionate group sizes.

In this review, only one patient out of 102 patients receiving opioid analgesics post operatively had to return to the operating room. Concerns with opioids typically include obscuring the clinical exam to the point of missing a catastrophic event which could lead to further morbidity or mortality [1,3,5-7,11,15-17]. In our study, the patient who required repeat surgery demonstrated a change in mental status that was identified despite the use of opioids. Therefore, it is possible to administer opioids to assist with pain control without obscuring the neurologic exam to the degree of missing a surgical lesion or causing an adverse event which is consistent with multiple studies [3-5]. Having a low threshold for repeat imaging can allow for the use of opioids in post craniotomy pain management. While pain is largely subjective, the physiologic consequences are still evidenced through the potential tachycardia, tachypnea, and hypertension, all of which can contribute to post-operative complications [13]. Therefore, pain is an important factor in post-operative management and opioid use should not be automatically eliminated from the post-operative plan.

## Conclusions

No statistical difference was found in pain scores in post-operative days one through three of patients who underwent craniotomy for emergent (within 24 hours of diagnosis) and non-emergent reasons when they were able to communicate a pain score either verbally or in writing. In our series, pain was reported verbally postoperatively, therefore, emergent cases represented in this patient population likely only included minor mechanisms of injury since they were able to be extubated postoperatively and there was a disproportionate amount of non-emergent craniotomies compared to emergent craniotomies for data analysis limiting the generalizability of findings.

## References

[1] Remy C, Marret E, Bonnet F (2005). Effects of acetaminophen on morphine side-effects and consumption after major surgery: meta-analysis of randomized controlled trials. Br J Anaesth.

[2] Williams DL, Pemberton E, Leslie K (2011). Effect of intravenous parecoxib on post-craniotomy pain. Br J Anaesth.

[3] Morad AH, Winters BD, Yaster M (2009). Efficacy of intravenous patient-controlled analgesia after supratentorial intracranial surgery: a prospective randomized controlled trial. J Neurosurg.

[4] Na HS, An SB, Park HP (2011). Intravenous patient-controlled analgesia to manage the postoperative pain in patients undergoing craniotomy. Korean J Anesthesiol.

[5] Stoneham MD, Cooper R, Quiney NF, Walters FJ (1996). Pain following craniotomy: a preliminary study comparing PCA morphine with intramuscular codeine phosphate. Anaesthesia.

[6] Jones SJ, Cormack J, Murphy MA, Scott DA (2009). Parecoxib for analgesia after craniotomy. Br J Anaesth.

[7] Rahimi SY, Alleyne CH, Vernier E, Witcher MR, Vender JR (2010). Postoperative pain management with tramadol after craniotomy: evaluation and cost analysis. J Neurosurg.

[8] Nguyen A, Girard F, Boudreault D (2001). Scalp nerve blocks decrease the severity of pain after craniotomy. Anesth Analg.

[9] Türe H, Sayin M, Karlikaya G, Bingol CA, Aykac B, Türe U (2009). The analgesic effect of gabapentin as a prophylactic anticonvulsant drug on postcraniotomy pain: a prospective randomized study. Anesth Analg.

[10] De Benedittis G, Lorenzetti A, Migliore M, Spagnoli D, Tiberio F, Villani RM (1996). Postoperative pain in neurosurgery: a pilot study in brain surgery. Neurosurgery.

[11] Rahimi SY, Vender JR, Macomson SD, French A, Smith JR, Alleyne CH (2006). Postoperative pain management after craniotomy: evaluation and cost analysis. Neurosurgery.

[12] Kaur A, Selwa L, Fromes G, Ross DA (2000). Persistent headache after supratentorial craniotomy. Neurosurgery.

[13] Mordhorst C, Latz B, Kerz T (2010). Prospective assessment of postoperative pain after craniotomy. J Neurosurg Anesthesiol.

[14] Kim YD, Park JH, Yang SH (2013). Pain assessment in brain tumor patients after elective craniotomy. Brain Tumor Res Treat.

[15] Saha P, Chattopadhyay S, Rudra A, Roy S (2013). Pain after craniotomy: a time for reappraisal?. Indian J Pain.

[16] Gottschalk A, Yaster M (2007). Pain management after craniotomy. Neurosurg Q.

[17] Haldar R, Kaushal A, Gupta D, Srivastava S, Singh PK (2015). Pain following craniotomy: reassessment of the available options. BioMed Res Int.

[18] de Gray LC, Matta BF (2005). Acute and chronic pain following craniotomy: a review. Anaesthesia.

